# A Multicentre Study of Prevalence and Risk Factors of Diabetic Retinopathy in Children and Young People With Type 1 Diabetes in the United Kingdom

**DOI:** 10.1111/dom.71316

**Published:** 2026-09-08

**Authors:** Darren J. C. Toh, Satish Hulikere, Rabia Mufti, Mary Masoud, Alison Timmis, Ellie Pearce, Pooja Sachdev, Iqra Zameer, Swathi Upadrasta, Ramneek Kaur Manku, Astha Soni, Sze May Ng

**Affiliations:** ^1^ University of Cambridge Cambridge UK; ^2^ North Cheshire and Mersey NHS Foundation Trust Warrington UK; ^3^ Mersey and West Lancashire Teaching Hospitals Ormskirk UK; ^4^ Countess of Chester Hospitals NHS Trust Chester UK; ^5^ University of Nottingham Nottingham UK; ^6^ Sheffield Children's Hospital NHS Trust Sheffield UK; ^7^ Edge Hill University Ormskirk UK

**Keywords:** diabetic retinopathy, glycaemic control, risk prediction, type 1 diabetes

## 1 Background

Type 1 Diabetes Mellitus is a metabolic condition that affects the body's ability to regulate blood glucose concentrations because the body cannot produce insulin. One of its complications is diabetic retinopathy (DR), an ophthalmic microvascular condition categorised into background retinopathy (R1) involving initial microaneurysms and retinal haemorrhage, pre‐proliferative retinopathy (R2) which involves venous beading and intraretinal microvascular abnormalities, and proliferative retinopathy (R3) characterised by retinal neovascularization [1, 2]. Commonly found risk factors include glycaemic control, duration of diabetes and hypertension, but many other factors have been found to be associated with DR as well [2, 3, 4, 5, 6, 7, 8, 9, 10, 11, 12].

While several studies have reported on the prevalence and risk factors of DR, recent data focusing solely on children and adolescents with T1D is limited, particularly in the United Kingdom, where the most recent data is from 2011 to 2019 [3]. There is limited data reflecting on possible associations between diabetes technologies and DR, such as if there is a relationship with the method of insulin administration (hybrid closed loop system, pump, multiple daily injection), especially with the now widespread use of continuous glucose monitoring and hybrid closed‐loop systems. Updated multicentre data are therefore needed to establish whether the burden and determinants of DR have changed in routine paediatric practice and to inform the emphasis placed on glycaemic, blood‐pressure and technology‐related interventions. The aims of this study were to obtain updated information about DR in children and adolescents, and to identify clinical risk factors associated with the presence of DR within our cohort using a cross‐sectional study.

## 2 Methods

Children and young people (CYP) with type 1 diabetes mellitus were included from 6 paediatric diabetes centres in England, United Kingdom. Eligible patients were aged 12–18 years and were registered with a regional DR screening programme. Screening outcomes recorded between January 2024 and December 2025 were reviewed centrally, where patients with an available documented retinal screening result during the study period were eligible for inclusion.

Retinopathy status was classified as: no DR (R0), background DR (R1), pre‐proliferative DR (R2) and proliferative DR (R3), with maculopathy recorded as absent or present (M0/M1) where available. Retinopathy was defined as R1 or above. Variables measured included age at screening, sex, age at diagnosis, duration of diabetes in years, insulin regimen, mean HbA1c, mean clinic blood pressure, mean urine albumin‐creatinine ratio (ACR) and ethnicity. Insulin regimen was categorised as multiple daily injections (MDI) and pump therapy consisting of continuous subcutaneous insulin infusion (CSII) and automated insulin delivery (AID), based on the regimen in use at the time closest to screening. Lipidemic and inflammatory risk factors were not measured.

Data was analysed using Statistical Package for the Social Sciences version 21 (SPSS Inc., Chicago). Participants with missing data were excluded. Univariable tests were performed to identify significant risk factors, which were then included into a multivariable logistic regression to identify factors independently associated with DR.

The project was registered as an evaluation audit of anonymised data submitted to the national paediatric diabetes register under Diab&End/CA/2026‐27/02. The study used routinely collected data, and all participating centres were responsible for extracting anonymised data. Results were reported in aggregate form to minimise the risk of re‐identification.

## 3 Results

811 participants were included across the six paediatric diabetes centres (Warrington = 98, Ormskirk = 88, Chester = 58, Whiston = 88, Sheffield = 201, Nottingham = 278). Excluding patients with missing data, DR prevalence was 11.8% (*n* = 80 of 676). Out of these 80 patients, 79 were classified as background DR (R1) and 1 as pre‐proliferative DR (R2); no cases of proliferative retinopathy or maculopathy were identified. Significant risk factors were younger age at diagnosis (*p* = 0.013), longer duration of diabetes (*p* < 0.001), higher mean Hba1c in the last 48 months (*p* = 0.038) and higher mean systolic blood pressure (SBP) (*p* = 0.007). No other factors tested were significant (Table 1). Multivariate logistic regression was then performed using significant risk factors. The logistic regression (chi^2^ = 27.479, *R* ^2^ = 0.107, *p* < 0.001) showed that longer duration of diabetes (*p* = 0.002) and higher mean systolic pressure (*p* = 0.043) were independent predictors of DR (Table 2).

**TABLE 1:** Univariable tests of factors affecting diabetic retinopathy, using independent Student *t*‐test for continuous outcomes and Chi^2^/Fisher's exact value for categorical outcomes.

Variables		Presence of retinopathy (*n* = 80)	Absence of retinopathy (*n* = 596)	Confidence interval (95%)	Chi^2^/Fisher's exact value	*p*
Age in 2025 (years)		13.96 ± 4.43	13.80 ± 3.56	2.05 (−0.86, 1.19)		0.753
Age at diagnosis (years)		8.04 ± 3.44	9.43 ± 4.11	2.19 (−2.49, −0.3)		0.013
Duration of diabetes (years)		8.94 ± 4.32	6.47 ± 3.97	1.88 (1.53, 3.41)		< 0.001
Mean Hba1c over last 48 months (mmol/mol)		62.46 ± 19.65	57.66 ± 13.31	9.06 (0.28, 9.34)		0.038
Mean systolic blood pressure (mmHg)		120.80 ± 9.64	117.75 ± 9.41	4.45 (0.82, 5.27)		0.007
Mean diastolic blood pressure (mmHg)		67.20 ± 6.91	67.73 ± 7.46	3.49 (−2.28, 1.21)		0.549
Mean urine ACR (mg/mmol)		1.41 ± 1.53	2.40 ± 19.53	9.32 (−5.65, 3.67)		0.677
Gender	Male	51.9%	57.7%		0.972^a^	0.324
	Female	48.1%	42.3%			
Insulin Regimen	AID/CSII	87.3%	81.2%		1.785^a^	0.182
	MDI	12.7%	18.8%			
Ethnicity	White	75%	80.7%		3.828^b^	0.406
	Mixed/Multiple	7.5%	4.6%			
	Asian	6.3%	6.3%			
	Black/African	5.0%	2.4%			
	Other	6.3%	6.1%			

^a^ Chi^2^ test.

^b^ Fisher exact test.

**TABLE 2:** Multivariable logistic regression of factors affecting retinopathy.

Variables	*B*	Exp(*B*)	95% Cl for Exp(*B*)	*p*
Age at diagnosis (years)	0.044	1.045	0.947, 1.154	0.380
Duration of diabetes (years)	0.161	1.174	1.059, 1.302	0.002
Mean Hba1c over last 48 months (mmol/mol)	0.013	1.013	0.996, 1.031	0.145
Mean systolic blood pressure (mmHg)	0.031	1.031	1.001, 1.063	0.043

*Note:* Omnibus test of model coefficients: *N* = 676, Chi^2^ = 27.479, *p* < 0.001, Hosmer and Lemeshow test: Chi^2^ = 6.457, *p* = 0.596, *R* ^2^ = 0.107.

## 4 Conclusions

In this multicentre cross‐sectional study of CYP with T1D, the prevalence of DR recorded was 11.8%, comparable to the reported DR prevalence of 12% in England, Wales and Jersey by the National Paediatric Diabetes Audit (NPDA) 2024/2025 annual report [13].

From our multivariable model, longer duration of diabetes and higher mean SBP were the most significant risk factors for DR. Increased duration of diabetes is a well‐known risk factor for DR [2, 3, 4, 5, 6, 7, 8, 11], supported by our study, with implications for children diagnosed very young, who may have long diabetes durations while under paediatric care and so may benefit from earlier screening. Elevated SBP can indicate vascular risk, although it does not establish that lowering blood pressure directly prevents DR. Additionally, some studies have reported an association between DR and diastolic blood pressure (DBP), in contrast to our findings [3, 4, 9]. Both SBP and DBP are deserving of a prospective study to determine their effect on DR incidence. Gender, ethnicity and urine ACR were not associated with DR.

Unusually, while mean Hba1c was significantly correlated with presence of DR, it was not significant after accounting for other factors in the multivariable logistic regression, despite several studies finding it a risk factor for DR [2, 3, 4, 5, 6, 7, 8, 10, 11, 12]. This may have been due to confounding with other variables, the limited number of retinopathy events, or improvement in glycaemic control after earlier years of higher exposure. Lifetime Hba1c, which has been shown to be an independent predictor of DR [10], may be more appropriate to measure instead. Age at diagnosis was also associated with DR in univariable but not multivariable tests, which could result from confounding, low numbers of individuals with DR or collinearity with duration of diabetes.

There was a lack of a significant association between insulin regimen and DR in our study, whereas other studies have also observed pump therapy to be associated with both better glycaemic control and lower DR odds [2, 11]. There are a number of possible explanations: Most participants were using pump or closed‐loop therapy, leaving a comparatively small MDI group and limiting statistical power; retinal changes observed in 2024–2025 may also reflect glycaemic exposure accumulated before adoption of current devices. This could instead be interpreted as access to technology alone is insufficient.

The study is limited by its cross‐sectional, retrospective design and inclusion of six centres predominantly in northern England, which may limit generalisability and prevent conclusions about causality or the temporal relationship between risk factors and DR.

In conclusion, the prevalence of DR in CYP with T1D remains comparable with current UK national estimates despite advances in diabetes management. Diabetes duration and mean systolic blood pressure were the only independent predictors of DR in this cohort. These findings suggest the importance of earlier screening and intervention of diabetes and stricter glycaemic control. Prospective longitudinal studies would be beneficial to determine whether recent advances in diabetes management translate into reduced long‐term retinal complications.

## Funding

The authors have nothing to report.

## Conflicts of Interest

The authors declare no conflicts of interest.

## Data Availability

The data that support the findings of this study are not publicly available but are available from the corresponding author upon request.
